# Nursing Knowledge and Skills in Hidradenitis Suppurativa: A Multi-Centre Exploratory Study

**DOI:** 10.3390/nursrep15020040

**Published:** 2025-01-26

**Authors:** Lucía Vilanova-Trillo, Alba-Elena Martínez-Santos, Josefa-del-Carmen Fernández-De-La-Iglesia, Laura Salgado-Boquete, Ángeles Flórez

**Affiliations:** 1 Dermatology Department, Pontevedra University Hospital, SERGAS, 36003 Pontevedra, Spain; lucia.vilanova.trillo@sergas.es (L.V.-T.); angeles.florez.menendez@sergas.es (Á.F.); 2 Inflammatory, Pediatric and Oncological Dermatology (DIPO) Research Group, Galicia Sur Health Research Institute (IIS Galicia Sur), SERGAS-UVIGO, 36312 Vigo, Spain; 3 Faculty of Nursing and Podiatry, Health Sciences Department, University of A Coruña, 15403 Ferrol, Spain; 4 Faculty of Education Sciences, Department of Pedagogy and Didactics, University of Santiago de Compostela, 15782 Santiago de Compostela, Spain

**Keywords:** hidradenitis suppurativa, nurses, nursing, health knowledge, attitudes, practise

## Abstract

**Background/Objectives:** Hidradenitis suppurativa (HS) is an under-treated chronic inflammatory disease that affects approximately 0.40–2% of the population. Despite the competences nurses have in wound care, little is known about their management of HS lesions. The objectives of this study were to determine the knowledge and skills of primary care nurses regarding HS as well as to analyse the aspects in which these nurses need to strengthen their competences. **Methods:** A multicentre exploratory cross-sectional study was carried out in all seven healthcare areas of the Galician Health Service (N-W Spain) during 2021–2022. A questionnaire was developed for the project and subsequently piloted; from it, four dimensions were established. Additionally, sociodemographic data were collected. A total of 211 primary care nurses participated. **Results:** Both total and dimension scores were lower than 5 out of 10 points. The participants showed more knowledge in the Diagnosis and Clinical Course dimension (M = 4.59 out of 10). An older age correlated with greater knowledge regarding Diagnosis and Clinical Course (rho = 0.196; *p* = 0.006). **Conclusions:** These findings highlight the importance of developing evidence-based continuous training in the management and detection of the disease, while reducing the consequences of HS on patients’ quality of life.

## 1. Introduction

Hidradenitis suppurativa (HS) is a chronic inflammatory disease that affects the terminal follicular epithelium of the apocrine glands. Clinically, it is defined by recurrent episodes of painful nodules, draining sinus tracts, or purulent abscesses, typically in intertriginous areas [1,2]. Although the aetiology remains unclear, it is considered a multifactorial disease where genetic, immunological, and environmental factors play a key role [2,3].

The diagnosis is exclusively clinical, so severity and clinical evolution may be difficult to assess.

A recent meta-analysis revealed that HS affects approximately 0.40–2% of the population [4], although the disease continues to be underdiagnosed and undertreated [1]. Thus, a decade often elapses from the first symptoms to the diagnosis of the disease, which impacts greatly on patients’ quality of life while they see their symptoms trivialised and not addressed [5]. During this process, some patients suffer numerous and very limiting symptoms regarding their wounds that affect their wellbeing, such as pain, pruritus, malodour, and suppuration, among others [6]. The greater intensity of one of these signs or symptoms was correlated with a worse quality of life, often including sexual anguish, anxiety, depression, and sleep problems [6]. Moreover, HS has been significantly associated with the presence of comorbidities [1].

Although the treatment is aimed at HS remission and minimising the sequelae [2,5], early detection, non-pharmacological interventions, and wound care are essential to reduce the loss of quality of life [2,7,8]. Thus, it is necessary to ensure interdisciplinary management based on the latest available evidence [1,9]. Primary care nurses, as members of the healthcare team and due to their role and skills, are crucial in the non-pharmacological treatments, such as wound care and lifestyle behaviour changes based on weight reduction, smoking cessation, or pain management, among others [8,10,11,12,13].

Despite this, nurses and other health professionals still face numerous challenges related to knowledge, attitudes, and practises regarding the disease [9,14]. In fact, patients have expressed barriers to access to care due to the difficulty in accessing professionals who know the disease, verbalising their intention to contribute to the healthcare recognition of HS [9,15,16].

Although HS is an undertreated disease unknown for many nurses [1,13], its prevalence is like that of other inflammatory dermatological diseases such as psoriasis [17], in which when nurses are well trained, and they manage to improve the quality of life of patients [18] while reducing wait times and increasing access to care [19]. Furthermore, although HS patients highlight the lack of training of health professionals outside of dermatology and how this influences the monitoring of their disease [9], little is known about the real skills of primary care nurses, who are usually responsible for wound care and the monitoring of chronic patients. Thus, based on the prevalence of the disease and the general lack of knowledge, all nurses may encounter incorrectly treated cases in primary care consultations, which could be minimised with adequate knowledge. Therefore, the aims were as follows: (1) to determine the knowledge and skills of primary care nurses on the diagnosis, risk factors, nursing care, and wound care of HS and (2) to analyse in which aspects these nurses need to strengthen their skills.

Based on these aims, we hypothesise that due to the limited evidence (mainly reported by patients with HS), low levels of knowledge and skills are expected among primary care nurses. However, it is anticipated that some factors related to experience (such as education level and professional category) may influence these outcomes.

## 2. Materials and Methods

### 2.1. Research Design

A multicentre exploratory cross-sectional study was carried out on the seven health areas of the Galician Health Service (north-west Spain), which is a regional public health system. To ensure adequate data reporting, the Strengthening the Reporting of Observational Studies in Epidemiology (STROBE) statement was used [20].

### 2.2. Participants and Data Collection

The study was performed during February 2021 and February 2022. Eligible participants were (1) registered nurses or family and community nurse specialists who carried out their care work in one of the seven health areas of the region and (2) accepted to participate after giving implicit consent in the questionnaire. Participants were recruited in all primary care centres in the region; therefore, no sampling procedure was utilised. The questionnaire was distributed electronically through corporate email and face-to-face to try to access nurses of all ages, who took an average of 20 min to complete it. The Microsoft Forms^®^ application, integrated into the Office 365^®^ package, was used to design and distribute the questionnaire. This application was chosen due to its user-friendly interface, accessibility across different devices, and built-in features to ensure data anonymity and confidentiality. Additionally, its compatibility with the institutional Office 365^®^ ecosystem allowed the seamless dissemination of the survey and facilitated data collection in a secure and organised manner. To calculate the necessary and significant sample size, we began with a target population of 2551 primary care nurses. Based on these data, with a level of confidence (1 − α) of 95%, a margin of error (d) of 5%, and a proportion or estimated percentage level of high performance (p) of 15%, 182 valid questionnaires were deemed necessary.

### 2.3. Instrument

An ad hoc questionnaire was developed for the project to assess the nurses’ socio-demographic and professional characteristics, as well as knowledge and skills on the diagnosis, risk factors, nursing care, and wound care of HS. For the design of the instrument, an exhaustive bibliographic review was carried out by 2 nurses and 2 dermatologists from the Hidradenitis Unit of the Dermatology Service of the University Hospital of Pontevedra (north-west Spain). Each of the authors selected the most important data following the review findings and their clinical experience in the management of HS. Researchers then elaborated the questionnaire based on the results of a national consensus guideline updated at design time [21]. Subsequently, they piloted it among the nurses of the same institution (different services) to ensure the correct reading comprehensibility and legibility of the question and answers.

The final questionnaire consisted of different multiple-choice questions related to the following 4 dimensions: Diagnosis and Clinical Course, Risk Factors and Triggers, Nursing Care, and Wound Care (Table 1). Cronbach’s alpha index with the sample of the present study revealed a good internal consistency (0.821), confirming the reliability and internal consistency of the instrument.

### 2.4. Data Analysis

Statistical analyses were performed using SPSS 28.0.1.1. (IBM Inc., Armonk, NY, USA). Descriptive analyses (frequencies, percentages, means (Ms), and standard deviations (SDs)) were obtained to describe the sample and values obtained in the ad hoc questionnaire. To provide a more exhaustive perspective on the participants’ stronger and weaker points, they were categorised as low performers (scores lower than the mean minus 1 standard deviation), average performers (scores between low performers and high performers), and high performers (scores higher than mean plus 1 standard deviation) in the total score and the dimensions scores. To summarise the results of the test, a sum of the items corresponding to the total questionnaire and for each dimension separately were obtained. The Kolmogorov–Smirnov test was used to verify the assumption of normality, where variables did not follow a normal distribution. Thus, non-parametric tests were carried out to contrast the effect of gender and contract type on the results. Additionally, non-parametric bivariate correlations (Spearman correlations) were obtained between the results of the questionnaire and Age and Work experience. Finally, to compare the level of the participants in the subtests (dimensions), scores were normalised on a 10 points basis for each dimension and compared using the Friedman test, followed by a posteriori pairwise comparison adjusted to Bonferroni correction. A *p* value < 0.05 was considered the nominal threshold for statistical significance.

### 2.5. Ethical Considerations

The study protocol was approved by the Regional Research Ethics Committee of Pontevedra-Vigo-Ourense (Approval number 2020/335). The information about the nature, purposes, and research team was included on the cover sheet of the questionnaire. The participation was voluntary, and data were processed anonymously. Therefore, implied consent was obtained according to current European and national regulations on data protection, where participants confirmed they had comprehensively read and understood the study, marking the corresponding statement at the beginning of the questionnaire. Without marking this statement, the questionnaire could not be completed.

## 3. Results

### 3.1. Sample Characteristics

Of the total population (*N* = 2551), 220 questionnaires were received, of which 9 were discarded due to being incompletely filled out. Therefore, the final sample consisted of 211 primary care nurses from seven health areas of the Galician Health Service who work in primary care, reaching a significant sample based on the population studied (response rate 8.3%). Of the total, 87.0% were females and 13.0% males. The average age was 44.2 years (SD = 12.76), with an average work experience of 21.9 years (SD = 13.0). Regarding the type of contract, 58.8% had a permanent contract, and 41.2% had a temporary one.

### 3.2. Descriptive Analyses

Table 1 shows the different dimensions and items of the nursing knowledge questionnaire on HS, including the possible answers (in italics) and the percentage of success in each of the questions. Using the normalised scores, the descriptive statistics showed that in all cases (total score, dimensions scores) the scores were lower than 5 out of 10 points. Thus, the total score average was 4.19 (SD = 1.68), where the Diagnosis and Clinical Course dimension received an average of 4.59 (SD = 2.07), Nursing Care an average of 3.92 (SD = 2.61), Risk Factors and Triggers an average of 3.70 (SD = 2.18), and the dimension related to the Wound Care obtained an average of 3.50 (SD = 1.73).

The distribution of the participants in the different scores according to their performance (low, average, and high performers) showed that 20.4% nurses displayed a global high performance (see Table 2 for more details).

In addition, only 5.0% of the participants answered that they had specific training in HS, and 94.1% of the sample said that they would like to receive training in HS care. Related to these, most of the participants reported a preference for an online training programme (60.1%), whereas 9.6% preferred onsite training and 11.7% a blended training model.

### 3.3. Statistical Contrasts

Regarding the effect of sex on the results of the questionnaire, the Mann–Whitney U tests have not found significant effects on the total score (U = 1958.5; *p* = 0.096) and the score for the four different dimensions (Diagnosis and Clinical Course: U = 2147.5; *p* = 0.307; Risk Factors and Triggers: U = 2176.5; *p* = 0.350; Nursing Care: U = 2095.0; *p* = 0.226; Wound Care: U = 2009.0; *p* = 0.123).

Regarding the effect of contract type on the results of the questionnaire, the Kruskal–Wallis H tests did not reveal any significant effects, either on the total score (H = 1.826; *p* = 0.401) or on the dimensions (Diagnosis and Clinical Course: H = 4.094; *p* = 0.129; Risk Factors and Triggers: H = 0.293; *p* = 0.864; Nursing Care: H = 0.385; *p* = 0.825; Wound Care: H = 1.200; *p* = 0.549).

The non-parametric Spearman tests revealed a weak, although significant correlation between Age and Diagnosis and Clinical Course dimension scores (rho = 0.196; *p* = 0.006). Thus, older age correlated with greater knowledge in this regard. No other significant correlations were detected.

Finally, the Friedman test showed significant differences between the z-scores of the different dimensions of the questionnaire (c2 = 66.972; *p* ≤ 0.001); scores in Diagnosis and Clinical Course dimension (4.59) were larger than scores in the Risk Factors and Triggers dimension (3.70; *p* ≤ 0.001), the Nursing Care dimension (3.92; *p* ≤ 0.001), and the Wound Care dimension (3.50; *p* ≤ 0.001).

## 4. Discussion

Interest in the research on and care of HS has increased exponentially in recent years [22]. This study complements the little evidence there is on the competences of primary care nurses in HS. Thus, further training, if made available to nurses, could be of enormous benefit to patients and the health system.

Wounds, including those caused by HS, are a major public health problem for the nursing community. Thus, poor care has important implications for healthcare systems and patients [23]. Considering that both our specific study on HS and the study by Ielapi et al. (2022) [23] on chronic wounds indicate a need for training, the importance of continuing to conduct studies that show the gaps in knowledge and skills should be emphasised to propose specific measures to improve the quality of care.

In our country, primary care nurses usually play a key role in the management of chronic patients, especially those who require wound care and comorbidity management [24,25]. Despite the role of nursing worldwide, we have not located any articles that study nurses’ knowledge regarding HS in any healthcare context.

In addition to topical and systemic therapies, both conventional and innovative, the wound care, lifestyle modification, and symptom control can improve wellbeing at any stage when appropriately applied [7,11,26]. In this vein, to ensure high-quality care, a wide variety of evidence-based approaches should be used in routine clinical care [22], following protocols if it is necessary [11]. Despite having clearly established competencies in other skin diseases such as dermatitis, psoriasis, or ulcers of various aetiology [27,28,29], as far as we know, in international consensus documents nurses rarely appear with specific functions. Due to the complexity of HS and the management of health system resources, it is essential to establish interdisciplinary work groups [30]. Thus, wound care specialist nurses could be part of a panel of HS healthcare providers, developing their work in wound care and health education (self-care as well as optimal management of comorbidities) [31,32].

We have shown that the Diagnosis and Clinical Course dimension is where both nurses and, concretely, older nurses, had more knowledge, despite this not being their competence. This could be related to the lack of research from a nursing perspective, which drives these professionals to resort to clinical approaches. Despite this, it would be necessary to investigate why differences only occur in relation to age, and not to other factors such as work experience in primary care. This finding may be relevant since we are in an environment where there are no reference primary care nurses in HS. Also, it is known that HS takes years to be treated adequately under diagnosis and that the so-called “window of opportunity” is related to the prognosis of the disease by reducing times and lesions [5,33].

Moreover, our study has found that primary care nurses need to improve both their knowledge and skills in all the dimensions of HS studied in this research, particularly regarding Wound Care, this being a dimension where nursing care is developed autonomously [26]. This could be due to the difficult and highly specific management of the disease, as it is very different from other chronic wounds. In this regard, it is necessary to consider this lack of training in the high importance that patients attach to the recognition of the impact that the disease has on the suffering and stigma [15,32]. Thus, greater awareness of the disease is essential for them [11] and the difficulty in accessing knowledgeable healthcare professionals is one of their greatest needs [9].

In this vein, the quality of life of HS patients is highly affected [1,34] and some of them report a lack of confidence in nurses’ knowledge and little empathy on their part [35]. To this end, it is important that nursing attention is in line with the revolutionary medical and surgical management of HS in recent years [26]. Also, patients affected by chronic wounds demand more research and therapies based on dressings [10]. Thus, well-qualified primary care nurses could enhance the organisational culture of health systems [36], while increasing the well-being of patients affected by HS and responding to their demands.

Thus, our findings suggest that focused training in HS wound care could empower nurses to autonomously deliver interventions that directly address patient needs. For example, providing detailed guidance on hygiene practises, pain management, and self-care routines could help reduce the stigma and psychological burden associated with HS. Other interventions could include the earlier detection of lesions, improved management of pain and associated symptoms, enhanced patient adherence to lifestyle modifications, and reduced time to effective intervention. For instance, well-trained nurses could identify disease exacerbations more rapidly, implement evidence-based wound care strategies, and support patients in managing comorbidities, ultimately enhancing their quality of life.

### Strengths and Limitations

To the best of our knowledge, this is the first study to explore nurses’ knowledge and skills regarding HS, showing how nurses can improve their training in specific aspects of the disease. Furthermore, an interdisciplinary team from the fields of nursing and dermatology has participated in the design and implementation of this research. Consequently, this study contributes to addressing the gap in the literature on HS. However, our research has certain limitations. The sample selection was intentional and in a single country, so a larger and international sample would be necessary to generalise findings. The questionnaire was developed ad hoc for this study at a specific time, which exposes the study to possible biases. Given the advances in therapy and knowledge of the disease, it will be necessary to update and select the questions and answers if the questionnaire is to be used currently. Also, knowledge questionnaires, due to their nature, cannot be strictly psychometrically validated, so an attempt has been made to systematise the creation and validation of content as much as possible.

## 5. Conclusions

This research has shown low knowledge in all dimensions studied, where less than a quarter of primary care nurses achieved a high performance despite being in contact with chronic wounds of a various nature. The dimension related to Wound Care obtained the lowest score despite including specific nursing competencies. Additionally, they have articulated their need for further training in HS. These findings highlight the importance of developing continuous training in the management and detection of the disease, particularly in topical therapy. Furthermore, well-trained nurses could provide evidence-based care in the management of symptoms and comorbidities, lifestyle modification, and topical care, improving quality of life from an early stage. Also, nurses, due to their role and competencies, could help recognise the disease by identifying its typical lesions, thus reducing referral and diagnosis times. Thus, quality interdisciplinary care could be provided in the face of an undertreated disease.

## Figures and Tables

**Table 1:** Dimensions and items of the nursing knowledge questionnaire on hidradenitis suppurativa, including the possible answers, correct answers, and the percentage of success in each of the questions.

Questions and Dimensions	Possible Answers	Correct Answer/s	Percentage of Success
**1. Diagnosis and Clinical Course dimension**			
1.1 The terms furuncle and hidradenitis suppurativa are synonyms	- True - False	- True	33.6%
1.2 Hidradenitis suppurativa is a chronic inflammatory skin disease. Indicate its clinical manifestations (select all that apply):	- Abscesses - Inflammatory nodules - Tunnels - I do not know	- Abscesses - Inflammatory nodules - Tunnels	52.1%
1.3 Indicate the anatomical areas in which the clinical manifestations of hidradenitis suppurativa appear (select all that apply):	- Armpits -Mammary and inframammary zone - Groins - External genitalia - Buttocks - Perianal area - I do not know	- Armpits - Mammary and inframammary zone - Groins - External genitalia - Buttocks - Perianal área	27.0%
1.4 The diagnosis of the disease is defined by the presence of painful subcutaneous nodules or abscesses located in the armpit, groin, buttocks, inframammary or perianal region in two or more outbreaks in the last 6 months	- True - False - I do not know	- True	66.4%
1.5 Hidradenitis suppurativa causes fever	- True - False - I do not know	- False	48.3%
1.6 Patients with hidradenitis suppurativa have adenopathies	- True - False - I do not know	- False	12.8%
1.7 Patients with hidradenitis suppurativa often have a family history of HS	- True - False - I do not know	- True	47.9%
1.8 Indicate which of the following options corresponds to the age of highest prevalence of hidradenitis suppurativa:	- Puberty (10–15 years) - 21–23 years - 60–65 years - Early childhood - I do not know	- 21–23 years	47.4%
1.9 According to the incidence by sex, hidradenitis suppurativa more frequently affects women	- True - False - I do not know	- True	59.7%
1.10 Indicate which of the following options corresponds to comorbidities associated with hidradenitis suppurativa (select all that apply):	- Inflammatory bowel disease (especially Crohn’s disease) - Spondyloarthropathy - Metabolic syndrome - Depression - Obesity - I do not know	- Inflammatory bowel disease (especially Crohn’s disease) - Spondyloarthropathy - Metabolic syndrome - Depression - Obesity	0.9%
1.11 Indicate which injury appears in the photograph (photography not included due to intellectual property):	- Inflammatory nodule - Tunnel - Abscess - I do not know	- Inflammatory nodule	73.5%
1.12 Indicate which injury appears in the photograph (photography not included due to intellectual property):	- Inflammatory nodule - Tunnel - Abscess - I do not know	- Abscess	71.1%
1.13 Indicate which are the injuries that appear in the photograph (photography not included due to intellectual property):	- Inflammatory nodule - Tunnel - Abscess - I do not know	- Tunnel - Abscess	55.5%
**2. Risk Factors and Triggers dimension**			
2.1 Tobacco is a triggering factor for hidradenitis suppurativa	- True - False - I do not know	- True	52.1%
2.2 Obesity is a trigger factor for hidradenitis suppurativa	- True - False - I do not know	- True	72%
2.3 The use of tight clothing is a trigger factor for hidradenitis suppurativa	- True - False - I do not know	- True	69.2%
2.4 The use of deodorants is a triggering factor for hidradenitis suppurativa	- True - False - I do not know	- True	43.6%
2.5 Bathing in the sea is a trigger factor for hidradenitis suppurativa	- True - False - I do not know	- False	2.8%
2.6 Hair removal is a trigger factor for hidradenitis suppurativa	- True - False - I do not know	- True	12.8%
2.7 Of the drugs listed below, select those that can cause repeated outbreaks of the disease:	- Contraceptives - Isotretinoin - Lithium - Non-steroidal anti-inflammatories - Antibiotics - I do not know	- Contraceptives - Isotretinoin - Lithium	6.2%
**3. Nursing Care dimension**			
3.1 Indicate which of the following options corresponds to the objective of the nursing consultation for patients with hidradenitis suppurativa (select all that apply):	- Give the patients as much information as possible about their pathology - Teach habits that prevent the appearance of outbreaks or exacerbations of the pathology - Carry out an adequate control of their quality of life - Early detection of other comorbidities associated with the pathology - Carry out the appropriate cures according to the standard defined for each type of injury - I do not know	- Give the patients as much information as possible about their pathology - Teach habits that prevent the appearance of outbreaks or exacerbations of the pathology - Carry out an adequate control of their quality of life Early detection of other comorbidities associated with the pathology - Carry out the appropriate cures according to the standard defined for each type of injury	56.1%
3.2 Indicate which of the following options corresponds to the procedures that should be performed on a patient with hidradenitis suppurativa in a nursing office (select all that apply):	- Temperature Record - Record of Blood Pressure and Heart Rate - Calculation of Body Mass Index (BMI) - Skin exploration (observe and palpate lesions) and record the evolution of the lesions - Obtaining the Quality of Life Scale - Obtaining the VAS Scale on pain and pruritus - Obtaining the Psychological Distress Questionnaire - Information about the disease and healthy lifestyle habits (verbal and written) - I do not know	- Temperature Record - Record of Blood Pressure and Heart Rate - Calculation of Body Mass Index (BMI) - Skin exploration (observe and palpate lesions) and record the evolution of the lesions - Obtaining the Quality of Life Scale - Obtaining the VAS Scale on pain and pruritus - Obtaining the Psychological Distress—Questionnaire - Information about the disease and healthy lifestyle habits (verbal and written)	24.2%
3.3 Indicate the correct option in relation to the professional who must carry out the cures of the lesions in a patient with hidradenitis suppurativa:	- They should always be performed by a physician - They should always be performed by the nurse - They can be performed by the physician or by the nurse - I do not know	- They can be performed by the physician or by the nurse	66.8%
3.4 Of the following options, indicate which are the hygiene rules that a patient with hidradenitis suppurativa must follow (select all that apply):	- Wash daily with antiseptic soap as many times as you need to eliminate the bad smell that the lesions give off - Wash with mild, non-rub soap no more than twice a day - Deodorants should not be used - I do not know	- Wash with mild, non-rub soap no more than twice a day - Deodorants should not be used	31.8%
3.5 Point out which are the preventive measures that should be actively recommended to all patients with hidradenitis suppurativa. (select all that apply):	- Give up smoking or, at least, reduce consumption - Avoid being overweight - Waxing with depilatory cream or shaving - Wear preferably cotton clothing - Laser hair removal - Do physical exercise - I do not know	- Give up smoking or, at least, reduce consumption - Avoid being overweight - Wear preferably cotton clothing - Laser hair removal - Do physical exercise	29.9%
3.6. Of the psychological symptoms listed below, please select those most frequently experienced by patients with hidradenitis suppurativa (select all that apply):	- Stress - Irritability - Low self-esteem - Sexual anguish - Social stigma - None. Hidradenitis suppurativa is a disease that has no psychological implications - I do not know	- Stress - Irritability - Low self-esteem - Sexual anguish - Social stigma	32.2%
3.7 Of the following options, indicate the behaviours that a patient with hidradenitis suppurativa may present to which special attention should be paid in the nursing consultation (select all that apply):	- Feeling sad - Irritability - Negativity - Suicidal ideation - Request for psychological or social care - None. Hidradenitis suppurativa is a disease that has no psychological implications - I do not know	- Feeling sad - Irritability - Negativity - Suicidal ideation - Request for psychological or social care	32.7%
**4. Wound care dimension**			
4.1 Indicate the correct option in relation to the treatment of a nodule (inflammatory or non-inflammatory) of a patient diagnosed with hidradenitis suppurativa:	- Should always be drained - Only inflammatory nodules should be drained - Do not drain. Treatment with topical antibiotic and intralesional corticosteroid infiltration should be performed - I do not know	- Do not drain. Treatment with topical antibiotic and intralesional corticosteroid infiltration should be performed	41.7%
4.2 Indicate the correct option in relation to the treatment of an abscess in a patient diagnosed with hidradenitis suppurativa:	- They should always be drained and prescribe topical antibiotic treatment on demand and systemic - They should only be drained if it causes disabling pain on examination and, if required, prescribe topical and systemic antibiotic treatment on demand - They should never be drained. Topical antibiotic treatment will be prescribed on demand and systemic - I do not know	- They should only be drained if it causes disabling pain on examination and, if required, prescribe topical and systemic antibiotic treatment on demand	51.2%
4.3 Indicate if this procedure to drain an abscess is correct: 1. Administer local anesthetic. 2. Make an incision with a scalpel. 3. Drain the contents (usually not purulent). To do this, open the lesion slightly with dissection forceps, Axon forceps, or mosquito forceps. 4. Leave a cotton gauze pad in place. 5. Optionally, apply a topical antibiotic (fusidic acid), especially if the patient is not going to take oral antibiotics. 6. Optionally, add a charcoal and/or silver disc to absorb odour. 7. Place an absorbent dressing that keeps the area dry, that adapts to the anatomical region, with a good adhesive, is non-irritating, and prevents odour.	- True - False - I do not know	- True	72%
4.4 A patient diagnosed with hidradenitis suppurativa who presents fistulas should be followed up in Primary Care, prescribing topical and systemic antibiotic treatment and pain control measures. You must make an appointment with the nursing office to be informed about healthy lifestyle habits and guidelines for self-care for injuries and specific cures for injuries	- True - False - I do not know	- False	4.3%
4.5 The action plan for a fistula consists of irrigating the inside of the fistula with physiological saline to clean any possible purulent material, cleaning the external area with antiseptic soap diluted with saline and placing a dressing (absorbent and adaptable)	- True - False - I do not know	- True	75.8%

**Table 2:** Primary care nurses’ knowledge of different dimensions of HS.

Dimension	Low Performance	Average Performance	High Performance
Diagnosis and Clinical Course	12.3%	42.2%	45.5%
Risk Factors and Triggers	23.7%	44.1%	32.2%
Nursing Care	30.8%	31.8%	37.4%
Wound Care	20.4%	59.2%	20.4%
Total	18.5%	61.1%	20.4%

## Data Availability

The raw data supporting the conclusions of this article will be made available by the authors on request.

## Public Involvement Statement

No public involvement in any aspect of this research.

## Guidelines and Standards Statement

This manuscript was drafted against the STROBE Checklist [19] for cross-sectional studies.
